# Impact of aerobic exercise on levels of IL‐4 and IL‐10: results from two randomized intervention trials

**DOI:** 10.1002/cam4.836

**Published:** 2016-08-03

**Authors:** Shannon M. Conroy, Kerry S. Courneya, Darren R. Brenner, Eileen Shaw, Rachel O'Reilly, Yutaka Yasui, Christy G. Woolcott, Christine M. Friedenreich

**Affiliations:** ^1^ Cancer Prevention Institute of CaliforniaFremontCalifornia; ^2^ University of California at DavisDavisCalifornia; ^3^ Faculty of Physical Education and RecreationUniversity of AlbertaEdmontonAlbertaCanada; ^4^ Department of Cancer Epidemiology and Prevention ResearchCancerControl AlbertaAlberta Health ServicesCalgaryAlbertaCanada; ^5^ Departments of Oncology and Community Health SciencesCumming School of MedicineUniversity of CalgaryCalgaryAlbertaCanada; ^6^ School of Public HealthUniversity of AlbertaEdmontonAlbertaCanada; ^7^ Departments of Obstetrics and Gynaecology and PediatricsDalhousie UniversityHalifaxNova Scotia, Canada

**Keywords:** Aerobic exercise, anti‐inflammatory markers, breast cancer, randomized controlled trial

## Abstract

The mechanisms whereby regular exercise reduces chronic inflammation remain unclear. We investigated whether regular aerobic exercise alters basal levels of interleukin (IL)‐10 and IL‐4 in two randomized trials of physical activity. The Alberta Physical Activity and Breast Cancer Prevention Trial (ALPHA, n = 320) and the Breast Cancer and Exercise Trial in Alberta (BETA, n = 400) were two‐center, two‐armed randomized trials in inactive, healthy, postmenopausal women. Both trials included an exercise intervention prescribed five times/week and no dietary changes. In ALPHA, the exercise group was prescribed 225 min/week versus no activity in the controls. BETA examined dose‐response effects comparing 300 (HIGH) versus 150 (MODERATE) min/week. Plasma concentrations of IL‐10 and IL‐4 were measured at baseline, 6, and 12 months. Intention‐to‐treat (ITT) analysis was performed using linear mixed models adjusted for baseline biomarker concentrations. Circulating anti‐inflammatory cytokine levels decreased among all groups, with percent change ranging from −3.4% (controls) to −8.2% (HIGH) for IL‐4 and −1.6% (controls) to −7.5% (HIGH) for IL‐10. No significant group differences were found for IL‐4 (ALPHA *P* = 0.54; BETA *P* = 0.32) or IL‐10 (ALPHA *P* = 0.84; BETA *P* = 0.68). Some evidence for moderation of the effect of exercise by baseline characteristics was found for IL‐10 but not for IL‐4. Results from these two large randomized aerobic exercise intervention trials suggest that aerobic exercise does not alter IL‐10 or IL‐4 in a manner consistent with chronic disease and cancer prevention.

## Introduction

Chronic systemic inflammation has been implicated in the etiology and pathogenesis of cardiovascular disease 1, 2, 3, 4, type 2 diabetes 5, 6, 7, and cancer 8, 9, 10. Particularly, high circulating C‐reactive protein (CRP), as a nonspecific marker of systemic inflammation, is a putative risk factor for postmenopausal breast cancer 11, 12, 13. Substantial evidence from observational studies and randomized controlled exercise intervention trials (RCTs) collectively demonstrate the beneficial effects of physical activity on chronic inflammation through a reduction in circulating proinflammatory biomarkers 14, 15, 16, 17, 18, 19, 20. It is plausible that physical activity offers protection against chronic inflammation, and thereby chronic disease and breast cancer development, by exerting systemic anti‐inflammatory effects mediated through the production of myokines via contracting skeletal muscles with a subsequent increase in anti‐inflammatory cytokines 21, 22, 23, 24. Myokines mediate local and systemic metabolic and anti‐inflammatory effects as well as adaptations related to long‐term physical activity (chronic vs. acute bouts of exercise) [25, 26, 27]. Furthermore, exercise‐induced muscle‐derived cytokines have been found to inhibit mammary cancer cell growth in vitro 28.

The Alberta Physical Activity and Breast Cancer Prevention Trial (ALPHA) and the Breast Cancer and Exercise Trial in Alberta (BETA) are two RCTs studying the effects of physical activity on putative biomarkers of breast cancer risk in previously sedentary postmenopausal women. Previous analyses from these trials showed that moderate–vigorous intensity exercise reduces CRP, but found limited evidence of a reduction in other proinflammatory markers, including interleukin (IL)‐6 and tumor necrosis factor (TNF)‐*α* 18, 19. To the best of our knowledge, no RCT in healthy postmenopausal women has explored the effects of regular exercise on anti‐inflammatory cytokines. Two promising candidates are IL‐4 and IL‐10. Both have physiological roles in inhibiting the expression and release of proinflammatory cytokines 29, 30, 31, postulated to contribute to longevity 32 through the protection against cardiovascular disease death 33 and cancer 34, and demonstrated antitumor activity, specifically on breast cancer cells 35, 36, 37, 38, 39. Epidemiologic and exercise intervention studies, including RCTs, have shown that regular exercise, primarily aerobic modalities, increases basal levels of IL‐4 40 and IL‐10 40, 41, 42, 43, 44, 45, 46, 47, 48, 49. Yet, the majority of the studies had a number of limitations, including exercise interventions without a comparison/control group 41, 45, 49, observational design 42, 47, small sample size (i.e., <50 participants 41, 43, 44, 47, 49, or among patients or individuals with metabolic abnormalities (i.e., diabetes/metabolic syndrome 40, 45, 48, cardiovascular disease 43, 44, 49, or kidney disease [46]). To address the limitations of these studies and the lack of data from RCTs in healthy subjects, we investigated whether varying volumes of moderate intensity aerobic exercise (150, 225 and 300 min per week) in the two trials (ALPHA and BETA) increase circulating levels of IL‐4 and IL‐10.

## Materials and Methods

### Study population

ALPHA and BETA were both two‐centered (Calgary and Edmonton, Alberta), two‐armed, year‐long RCTs in postmenopausal, sedentary women that have been described in detail elsewhere [50, 51]. ALPHA included 320 women and was conducted between May 2003 and June 2006, and BETA included 400 women and was conducted between July 2010 and April 2013. Both study protocols were approved by the Alberta Cancer Research Ethics Committee, the Conjoint Health Research Ethics Board of the University of Calgary, and the Health Research Ethics Board of the University of Alberta and all participants provided signed informed consent to participate in these studies.

The eligibility criteria included: (1) resident of Calgary or Edmonton; (2) 50–74 years of age; (3) nonhormone replacement therapy user within the 12 months prior to enrollment; (4) body mass index (BMI) between 22–40 kg/m^2^; (5) <14 alcoholic drinks per week on average; (6) nonsmokers; (7) inactive; (8) able to do unrestricted or progressive physical activity as assessed by physician screening; (9) normal levels of cholesterol, fasting blood glucose (<7 mmol/L), thyroid‐stimulating hormone, and alanine aminotransferase; (10) cancer‐free; and (11) not on a weight loss program or planning to commence one. Postmenopausal status was defined as one of: natural cessation of menstrual periods for at least 24 months; bilateral oophorectomy; hysterectomy without bilateral oophorectomy and age ≥55 years; or hysterectomy without bilateral oophorectomy and age 50–54 years with a follicle‐stimulating hormone level >30 IU/L. Inactivity was defined as <90 min/week of exercise or if between 90 and 120 min/week, having a VO_2max_ < 34 mL/kg/min as assessed by a submaximal fitness test.

### Exercise intervention

In ALPHA, women were randomized to either a 1‐year aerobic exercise intervention of 225 min per week (*n* = 160) or to a control group, who were instructed to maintain their usual level of activity (*n* = 160). In BETA, women were randomized to either a moderate‐volume aerobic exercise intervention of 150 min per week (*n* = 200) or a high‐volume exercise intervention of 300 min per week (*n* = 200), based on Health Canada's Guidelines [52, 53] for aerobic activity and physical activity guidelines from the American Cancer Society [54, 55]. For all exercising participants, exercise sessions took place five times per week targeted at 70–80% heart rate reserve, with 3 days supervised by certified exercise trainers and 2 days of home‐based unsupervised sessions. Exercise adherence was monitored using weekly exercise logs. Heart rate monitors were worn to ensure that at least 50% of time in each exercise session was completed within the target heart rate zone. Methods to ensure adherence to exercise interventions have been previously described [50, 51]. In both trials, participants were asked not to change their normal dietary intake.

### Biomarker assays

Blood collected from each participant at baseline (60 mL), 6, and 12 months (40 mL). Blood draws were performed after a minimum 10‐h fast and complete abstinence from exercise and alcohol for 24 h. Aliquots of plasma were stored within 12 h of collection in **−**86°C freezer until the time of assay. Plasma samples were batched so that each batch contained: baseline and follow‐up samples for the same woman (in random order), an equal number of intervention and control bloods, and pooled quality assurance samples to estimate intra‐ and interbatch coefficients of variation (CV). Laboratory staff members were blinded to subject and quality control sample identities. The cytokines of interest were assayed at Eve Technologies (Calgary, AB, Canada) using the Bio‐Plex^®^ 200 system (Bio‐Rad Laboratories, Inc., Hercules, CA). The cytokines were multi‐plexed using a custom high‐sensitivity, 3‐plex assay for IL‐1ra, IL‐4, and IL‐10. The sensitivities for IL‐4 and IL‐10 ranged from 0.05 to 0.48 pg/mL. For ALPHA, the intra‐ and interbatch CV were 9% and 10%, respectively, for IL‐4, and 7% and 9%, respectively, for IL‐10. In BETA, the intra‐ and interbatch CV were both 9% for IL‐4, and 11% and 13%, respectively, for IL‐10. IL‐1ra was assayed, however, the assay yielded relatively high intra‐ and interbatch CVs (15% and 43%, respectively); thus, IL‐1ra results were excluded from the analysis.

### Statistical analysis

Both intention‐to‐treat (ITT) and per‐protocol analyses were performed, in addition to an analysis based on exercise adherence, using mean minutes of activity per week. The Spearman's rank‐order correlation was used to explore the relationship between cytokines (baseline and 12‐month change). The natural logarithm transformation was applied to normalize the distribution of IL‐4 and IL‐10 levels and the back‐transformed values are presented in the results. Participants were excluded for analysis if they did not provide a blood sample at each of the three time points (*n* = 18 for ALPHA and *n* = 16 for BETA) or their cytokine levels were above a threshold level (*n* = 5 for ALPHA and *n* = 1 for BETA for each IL‐4 and IL‐10) to remove extremely high levels. Due to the very limited number of missing samples, there was likely minimal bias as a result of removing those participants with incomplete follow‐up. For the ITT analysis, linear mixed models were used to evaluate intervention effects on anti‐inflammatory cytokine levels at 6 and 12 months, considered as repeated measures. The models were adjusted for baseline levels and included main effects of intervention, time, and their interaction term. Treatment effect ratios (TERs) were obtained as a geometric mean ratio for the exercise group over the control group (ALPHA) or the high‐volume over the moderate‐volume exercise group (BETA) from the mixed models. A TER greater than 1.0 is indicative of the exercise group (ALPHA) or high‐volume exercise group (BETA) having higher anti‐inflammatory cytokine values. Despite allocation of intervention groups by randomization, we evaluated potential confounding by examining the change in *β*‐coefficients with and without the potential confounder in the model. Through this assessment, we were able to confirm that baseline differences in dietary intake, cholesterol levels, arthritis, and use of nonsteroidal anti‐inflammatory drugs between randomization groups in ALPHA and BETA did not confound the intervention effects in either trial.

Potential moderation of the intervention effect by baseline characteristics was hypothesized a priori, as previously done analyses from ALPHA [56] and BETA 19. Moderation was evaluated using the value of the interaction term (*P*_heterogeneity_) between the randomization group assignment and each proposed moderator at baseline and the models used in the ITT analysis with the addition of the proposed moderator. Hypothesized moderators were assessed as continuous variables and included baseline levels of: fitness (VO_2_max), age, past year recreational physical activity, BMI, body fat percentage, IL‐4, and IL‐10. Subgroup analyses were performed based on the median for each variable, with the exception of BMI, which was separated into clinically meaningful groups of normal weight (<25 kg/m^2^), overweight (≥25–<30 kg/m^2^), and obese (≥30 kg/m^2^). Due to the association between adiposity and inflammation, potential mediation of the exercise intervention effect by changes in body weight, percent body fat, total body fat, and intra‐abdominal fat area was evaluated. Mediation was determined to be present if the TER changed after adjusting for the hypothesized mediator (adiposity change) and the *P*‐value for the hypothesized mediator was <0.05, as well as an effect of the adiposity change on the anti‐inflammatory marker level being present, after adjusting for the intervention assignment.

Per‐protocol analyses and adherence analyses were also performed using linear models. Among the exercisers, per‐protocol analyses included participants who had completed at least 80% of the exercise prescription: >180 min per week for the exercise group in ALPHA; and 120–150 min per week for the moderate‐volume group and >240 min per week for the high‐volume group in BETA. Thus, in the per‐protocol analysis, we excluded 53 exercisers in ALPHA and 75 moderate‐volume and 107 (107 for IL‐4 analysis or 106 for IL‐10) high‐ volume exercisers in BETA. In the adherence analyses, adherence categories were defined as <150 min per week, 150–225 min per week, and >225 min per week in ALPHA and <150 min per week, 150–250 min per week, and >250 min per week in BETA, based on the Canadian Physical Activity Guidelines of 150 min per week of exercise [52]. Inflammatory marker levels within the highest two adherence groups were tested against the lowest adherence group, and a trend test across adherence groups was performed using a linear model adjusted for baseline. Statistical tests were two‐sided, with a significance level set at *P *=* *0.05. All statistical analyses were performed using SAS software (Version 9.2; SAS Institute, Cary, NC).

## Results

**Figure 1:**
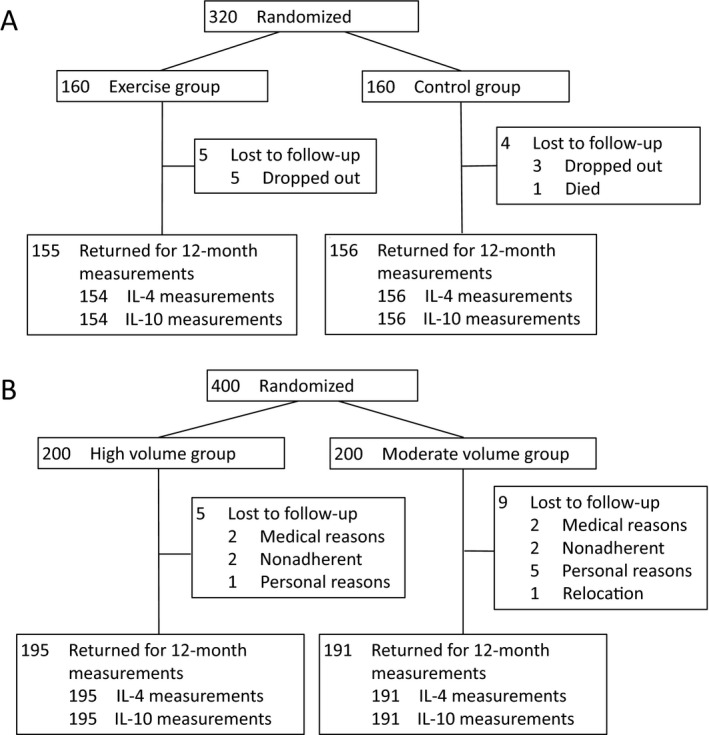
Randomization and follow‐up of participants in (A) the Alberta Physical Activity and Breast Cancer Prevention Trial and (B) the Breast Cancer and Exercise Trial in Alberta.

In ALPHA, 527 women met the initial inclusion criteria and of these, 320 women were randomized to either the exercise or control arm (Fig. 1). In BETA, of the 863 women who met the inclusion criteria, 400 women were randomized to either the high‐ or moderate‐volume exercise arm. In ALPHA, nine participants did not complete the trial, while 14 participants were lost to follow‐up in BETA, resulting in a 97% retention rate in both trials. We observed very similar baseline characteristics between randomization groups in each trial (Table 1). With the exception of the proportion of Caucasian participants in BETA, no imbalances were apparent between groups. The baseline IL‐4 and IL‐10 levels were slightly lower in BETA than in ALPHA. At baseline, IL‐4 ad IL‐10 were moderately correlated (*ρ *= 0.65 for ALPHA and *ρ *= 0.39 for BETA, *P *<* *0.01 for all), but were not correlated with age or BMI. Change in IL‐4 and IL‐10 over the 12‐months were weakly correlated (*ρ *= 0.35 for ALPHA and *ρ *= 0.13 for BETA, *P *≤* *0.01 for all). Changes in CRP were weakly correlated with changes in IL‐10 in BETA (*ρ *= 0.10, *P *=* *0.05), yet there was no evidence of a correlation with IL‐10 in ALPHA or with IL‐4 in both trials.

**Table 1:** Baseline characteristics of study participants from the Alberta Physical Activity and Breast Cancer Prevention Trial (ALPHA, *n* = 320) and the Breast Cancer and Exercise Trial in Alberta (BETA, *n* = 400)

Baseline characteristics	ALPHA		BETA	
	Exercisers (*n* = 160)	Controls (*n* = 160)	Moderate exercisers (*n* = 200)	High exercisers (*n* = 200)
	Mean ± SD	Mean ± SD	Mean ± SD	Mean ± SD
Age (years)	61.2 ± 5.4	60.6 ± 5.7	59.5 ± 5.1	59.4 ± 4.8
Body composition measurements				
BMI (kg/m^2^)	29.1 ± 4.5	29.2 ± 4.3	29.4 ± 4.4	29.1 ± 4.4
Intra‐abdominal fat area (cm^2^)^a^	101.4 ± 55.4	103.2 ± 56.0	133.4 ± 49.3	125.6 ± 50.8
Total body fat (kg)	30.9 ± 8.2	31.3 ± 8.6	31.0 ± 8.7	30.8 ± 8.6
Percent body fat	42.2 ± 4.9	42.4 ± 5.7	40.7 ± 5.9	40.5 ± 5.8
Alcohol intake (g/d)	4.4 ± 5.9	5.0 ± 7.6	5.3 ± 7.1	5.5 ± 8.5
Total energy intake (kcal/d)	1551.2 ± 598.7	1527.3 ± 535.0	1474.0 ± 541.4	1462.1 ± 588.2
Past year total physical activity (MET‐h/week)				
Total physical activity	114.2 ± 57.6	129.1 ± 77.9	96.4 ± 48.2	93.7 ± 44.1
Occupational activity	50.4 ± 49.1	52.2 ± 57.9	35.0 ± 34.5	36.4 ± 34.3
Household activity	52.9 ± 34.3	63.9 ± 53.5	50.3 ± 33.9	48.1 ± 32.7
Recreation activity	10.2 ± 11.8	12.1 ± 13.6	9.9 ± 13.6	8.5 ± 9.4
Maximal oxygen consumption (mL/kg/min)	27.1 ± 6.2	26.8 ± 6.0	26.8 ± 5.0	26.7 ± 5.3

	*n* (%)	*n* (%)	*n* (%)	*n* (%)
Full‐time employment	82 (55)	79 (51)	59 (30)	71 (36)
Education beyond high school	112 (70)	102 (64)	155 (78)	155 (78)
Married/common‐law	113 (71)	125 (78)	139 (70)	136 (68)
White/Caucasian	144 (91)	145 (91)	186 (93)	172 (86)
Ever used hormone therapy	75 (47)	71 (44)	55 (28)	62 (31)
Regular NSAID user	18 (11)	24 (15)	18 (9)	14 (7)
Regular statin user	25 (16)	18 (11)	25 (13)	21 (11)

	Median (IQR)	Median (IQR)	Median (IQR)	Median (IQR)
IL‐4 (pg/mL)	1.57 (0.82–2.11)	1.70 (0.95–2.42)	0.83 (0.41, 2.23)	0.92 (0.46, 2.03)
IL‐10 (pg/mL)	1.35 (0.81–1.90)	1.48 (0.81–1.87)	0.79 (0.49, 1.46)	0.76 (0.45, 1.42)

BMI, body mass index; IL‐4, interleukin‐4; IL‐10, interleukin‐10; IQR, interquartile range; NSAID, nonsteroidal anti‐inflammatory drug; SD, standard deviation.

a Different algorithms were used to calculate intra‐abdominal fat area from computerized tomography (CT) scans between ALPHA and BETA.

In the ITT analysis, we did not observe any significant differences in the 6 and 12 month levels of IL‐4 and IL‐10 between the exercise and control groups in ALPHA or the high‐ and moderate‐volume exercise groups in BETA (Table 2). Overall, circulating anti‐inflammatory cytokine levels decreased during the 1‐year exercise interventions among all groups, with percent change ranging from **−**3.4% (controls) to **−**8.2% (high‐volume exercisers) for IL‐4 and **−**1.6% (controls) to **−**7.5% (high‐volume exercisers) for IL‐10. All TERs were below the null value of 1.0, suggesting that the postintervention levels of circulating anti‐inflammatory cytokines were lower in the exercise group compared to the control group in ALPHA and in the high‐volume compared to the moderate‐volume exercise group in BETA.

**Table 2:** Intention‐to‐treat analysis of circulating anti‐inflammatory cytokine levels for exercisers and controls in the Alberta Physical Activity and Breast Cancer Prevention Trial (ALPHA) and high‐volume and moderate‐volume exercisers in the Breast Cancer and Exercise Trial in Alberta (BETA) at baseline, 6, and 12 months

	Baseline	6 months	12 months	*n*	Percent change from baseline to 12 months	TER of exercise/control or high/moderate (95% CI)^b^	Between‐group *P*
	Geometric mean (95% CI)^a^	Geometric mean (95% CI)^a^	Geometric mean (95% CI)^a^				
ALPHA							
IL‐4 (pg/mL)							
Exercisers	1.37 (1.23, 1.54)	1.34 (1.20, 1.49)	1.32 (1.18, 1.47)	150	**−**4.28	0.98 (0.92, 1.05)	0.54
Control	1.57 (1.40, 1.76)	1.52 (1.35, 1.71)	1.52 (1.35, 1.71)	147	**−**3.44		
IL‐10 (pg/mL)							
Exercisers	1.27 (1.13, 1.42)	1.25 (1.12, 1.41)	1.25 (1.11, 1.40)	150	**−**1.51	0.99 (0.91, 1.08)	0.84
Control	1.36 (1.22, 1.52)	1.35 (1.19, 1.52)	1.34 (1.19, 1.51)	147	**−**1.63		
BETA							
IL‐4 (pg/mL)							
High	0.82 (0.70, 0.97)	0.81 (0.69, 0.95)	0.76 (0.64, 0.89)	192	**−**8.24	0.94 (0.84, 1.06)	0.32
Moderate	0.89 (0.75, 1.05)	0.94 (0.79, 1.11)	0.83 (0.70, 0.99)	191	**−**6.32		
IL‐10 (pg/mL)							
High	0.82 (0.73, 0.92)	0.78 (0.70, 0.88)	0.76 (0.68, 0.85)	192	**−**7.53	0.98 (0.92, 1.06)	0.68
Moderate	0.88 (0.78, 0.99)	0.84 (0.75, 0.94)	0.82 (0.73, 0.93)	191	**−**6.35		

CI, confidence interval; IL‐4, interleukin‐4; IL‐10, interleukin‐10; TER, treatment effect ratio.

a Of the 310 ALPHA and 386 BETA participants who provided blood samples at any time point, we excluded those with IL‐4 or IL‐10 levels above a threshold for extremely high levels, specifically: 10 pg/mL for both IL‐4 (*n* = 5 excluded) and IL‐10 (*n* = 5 excluded) in ALPHA; and 50 pg/mL for IL‐4 (*n* = 1 excluded) and 35 pg/mL for IL‐10 (*n* = 1 excluded) in BETA. Participants (*n* = 2 for BETA and *n* = 8 for ALPHA) missing a blood sample at any time point were also removed.

b The TER was calculated based on a linear mixed model for each cytokine, adjusted for time and baseline value. The TER represents the adjusted ratio of geometric means for the exercise group over the control group (ALPHA) or the high‐volume exercise group over the moderate‐volume exercise group (BETA). A TER of <1.0 indicates lower anti‐inflammatory cytokine levels in the exercise group relative to the control group (ALPHA) or the high‐volume exercise group relative to the moderate‐volume exercise group (BETA) at 6 and 12 months; a TER greater than 1 indicates higher anti‐inflammatory markers in the exercise group (ALPHA) or the high‐volume exercise group (BETA); and a TER of 1.0 indicates no differences between groups.

Moderation of the effect of exercise by baseline characteristics was found for IL‐10, but not for IL‐4, with differential effects by physical fitness (*P *<* *0.01), age (*P *=* *0.07), BMI (*P *=* *0.04) and baseline IL‐10 levels (*P *=* *0.03) in ALPHA (Table 3) and physical fitness (*P *=* *0.02) and age (*P *<* *0.01) in BETA (Table 4). Positive TERs, suggesting higher postintervention levels of circulating IL‐10 in the exercise group compared to control group, were found in ALPHA among women who were more physically fit (VO_2_ max ≥27.5 mL/kg/min), aged ≤60 years, of normal weight (BMI < 25 kg/m^2^), and had higher IL‐10 levels at baseline. In contrast, significantly positive TERs within BETA, indicating higher postintervention levels of circulating IL‐10 in the high‐volume compared to the moderate‐volume exercise group, were found among women who were less physically fit (VO_2max_ < 27.2 mL/kg/min) and aged >60 years at baseline. Effects of the exercise intervention were not found to be mediated by changes in adiposity.

**Table 3:** Exercise intervention effects on inflammatory cytokines in the Alberta Physical Activity and Breast Cancer Prevention Trial (ALPHA), stratified by potential moderators

Potential moderator^a^	IL‐4^b^		IL‐10^b^	
Baseline level	*n* c	TER^d^	*n* c	TER^d^
Physical fitness (VO_2_max)				
<27.5 mL/kg/min	72/76	0.95 (0.86, 1.04)	73/75	0.91 (0.82, 1.01)
≥27.5	75/74	1.02 (0.93, 1.12)	74/75	1.09 (0.96, 1.23)
		*P* e = 0.66		*P* e < 0.01
Age				
≤60 years	73/68	1.05 (0.95, 1.15)	73/68	1.05 (0.92, 1.20)
>60	74/82	0.92 (0.84, 1.01)	74/82	0.94 (0.85, 1.04)
		*P* e = 0.38		*P* e = 0.07
Past year recreational activity				
<7.1 MET‐h/week	69/78	1.02 (0.93, 1.12)	70/78	1.02 (0.92, 1.13)
≥7.1	78/72	0.94 (0.85, 1.03)	77/72	0.96 (0.84, 1.09)
		*P* e = 0.29		*P* e = 0.28
BMI				
<25 kg/m^2^	28/32	1.03 (0.90, 1.18)	29/32	1.12 (0.88, 1.42)
25–<30	62/60	0.96 (0.86, 1.06)	61/61	1.01 (0.90, 1.14)
≥30	76/71	0.98 (0.87, 1.09)	76/72	0.91 (0.81, 1.02)
		*P* e = 0.45		*P* e = 0.04
Body fat percentage				
<42.2%	71/77	0.99 (0.90, 1.09)	72/77	1.02 (0.92, 1.13)
≥42.2	76/73	0.97 (0.89, 1.07)	75/73	0.97 (0.85, 1.10)
		*P* e = 0.40		*P* e = 0.26
Baseline IL‐4				
<0.50 pg/mL	71/79	1.04 (0.94, 1.15)		
≥0.50	76/71	0.92 (0.85, 1.00)		
		*P* e = 0.45		
Baseline IL‐10				
<0.36 pg/mL			71/78	1.09 (0.95, 1.24)
≥0.36			76/72	0.90 (0.82, 0.98)
				*P* e = 0.03

BMI, body mass index; CI, confidence interval; IL‐4, interleukin‐4; IL‐10, interleukin‐10 ; TER, treatment effect ratio.

a Level of potential moderator at baseline.

b Of the 310 participants who provided a blood sample at any time point, we excluded those with IL‐4 or IL‐10 levels above a threshold for extremely high levels, specifically: 10 pg/mL for both IL‐4 (*n* = 5 excluded) and IL‐10 (*n* = 5 excluded). Participants (*n* = 8) missing a blood sample at any time point were also removed.

c Number of exercisers/number of controls.

d The TER represents the adjusted ratio of geometric means for the exercise group over the control group. A TER of <1.0 indicates lower anti‐inflammatory cytokine levels in the exercise group relative to the control group at 6 and 12 months; a TER greater than 1 indicates higher anti‐inflammatory cytokine levels in the exercise group; and a TER of 1.0 indicates no differences between the groups.

e *P*‐value refers to the statistical significance of the interaction term between the exercise group and the potential moderator. All moderators were treated as continuous variables.

**Table 4:** Exercise intervention effects on inflammatory cytokines in the Breast Cancer and Exercise Trial in Alberta (BETA) , stratified by potential moderators

Potential moderator^a^	IL‐4^b^		IL‐10^b^	
Baseline level	*n* c	TER^d^	*n* c	TER^d^
Physical fitness (VO_2_max)				
<27.2 mL/kg/min	93/97	0.86 (0.73, 1.02)	93/96	1.11 (1.00, 1.23)
≥27.2	98/95	1.03 (0.89, 1.20)	98/96	0.88 (0.80, 0.97)
		*P* e = 0.76		*P* e = 0.02
Age				
≤60 years	108–119	0.90 (0.78, 1.04)	108/119	0.88 (0.80, 0.96)
>60	83/73	1.02 (0.85, 1.22)	83/73	1.16 (1.03, 1.31)
		*P* e = 0.90		*P* e < 0.01
Past year recreational activity				
<5.7 MET‐h/week	99/92	0.93 (0.80, 1.07)	99/93	0.92 (0.83, 1.03)
≥5.7	92/100	0.96 (0.81, 1.14)	92/99	1.04 (0.95, 1.15)
		*P* e = 0.83		*P* e = 0.52
BMI				
<25 kg/m^2^	38/41	0.96 (0.73, 1.27)	38/40	0.97 (0.81, 1.15)
25–<30	78/78	0.91 (0.77, 1.07)	78/78	0.95 (0.85, 1.05)
≥30	89/102	0.97 (0.80, 1.16)	98/96	1.04 (0.92, 1.17)
		*P* e = 0.81		*P* e = 0.72
Body fat percentage				
<40.3%	95/96	0.94 (0.80, 1.11)	95/96	0.95 (0.85, 1.05)
≥40.3	96/97	0.95 (0.81, 1.11)	96/96	1.03 (0.92, 1.14)
		*P* e = 0.63		*P* e = 0.92
Baseline IL‐4				
<0.90 pg/mL	97/94	1.01 (0.85, 1.21)		
≥0.90	94/98	0.91 (0.80, 1.04)		
		*P* e = 0.24		
Baseline IL‐10				
<0.76 pg/mL			93/96	1.00 (0.92, 1.10)
≥0.76			98/96	0.97 (0.86, 1.09)
				*P* e = 0.27

BMI, body mass index; CI, confidence interval; IL‐4, interleukin‐4; IL‐10, interleukin‐10; TER, treatment effect ratio.

a Level of potential moderator at baseline.

b Of the 386 BETA participants who provided blood samples at any time point, we excluded those with IL‐4 or IL‐10 levels above a threshold for extremely high levels, specifically: 50 pg/mL for IL‐4 (*n* = 1 excluded) and 35 pg/mL for IL‐10 (*n* = 1 excluded). Participants (*n* = 2) missing a blood sample at any time point were also removed.

c Number of exercisers/number of controls.

d The TER represents the adjusted ratio of geometric means for the exercise group over the control group. A TER of <1.0 indicates lower anti‐inflammatory cytokine levels in the high‐volume compared to moderate‐volume exercise group at 6 and 12 months; a TER greater than 1 indicates higher anti‐inflammatory cytokine levels in the high‐volume exercise group; and a TER of 1.0 indicates no differences between the groups.

e *P*‐value refers to the statistical significance of the interaction term between the exercise group and the potential moderator. All moderators were treated as continuous variables.

Similar with the ITT analysis, no postintervention effect on circulating IL‐4 or IL‐10 was found based on the per‐protocol analysis (Table S1) or adherence analysis (Table S2). This lack of effect was further demonstrated in Figure S1 that shows little change in the untransformed IL‐4 and IL‐10 levels over the 1‐year exercise interventions. In stratified analysis by adherence (<150 min per week, 150–225 min per week, and >225 min per week (ALPHA) or <150 min per week, 150–250 min per week, and >250 min per week (BETA)), no significant trends were found in either ALPHA or BETA across adherence groups (Table S2). Overall, there appeared to be a general decrease in circulating anti‐inflammatory cytokines levels during the 1‐year intervention, consistent with the ITT analysis.

## Discussion

To the best of our knowledge, ALPHA and BETA represent the first RCTs to examine the impact of regular moderate‐intensity aerobic exercise or the effect of aerobic exercise volume on anti‐inflammatory biomarkers in healthy, postmenopausal women. Collectively, we did not observe any significant effects of exercise on circulating levels of IL‐4 and IL‐10 after a 1‐year exercise intervention in ALPHA or BETA. Contrary to our hypothesis, we observed slightly lower levels of circulating IL‐4 and IL‐10 with aerobic exercise and with higher volume of exercise; yet the effects were not statistically significant.

Our findings do not support the notion that regular aerobic exercise leads to increased basal levels of IL‐4 and IL‐10. The lack of an effect in the present studies of a 1‐year, aerobic exercise intervention on circulating IL‐4 and IL‐10 may reflect an overall normal metabolic and inflammatory profile in healthy individuals, compared to those with chronic conditions, and thus less adaptive response to the exercise‐induced anti‐inflammatory effects in respect to enhanced basal levels. As reported elsewhere, the median baseline CRP levels for ALPHA 18 and BETA 19 were ≤1.90 mg/L among the four intervention groups, below the 3.0 mg/L threshold considered high risk for diabetes and cardiovascular disease [57, 58].

The RCTs 40, 43, 44, 48, 50, 60, 61 or intervention trials (all without a comparison/control group) 41, 45, 46, 49, 62 examining the effects of regular physical activity regimens on circulating anti‐inflammatory cytokines have been primarily conducted in patients or individuals with specific chronic diseases (i.e., diabetes or cardiovascular diseases). Most studies report an increase in levels of circulating anti‐inflammatory cytokines with regular exercise 40, 41, 43, 44, 45, 46, 48, 49. A year‐long RCT in diabetic patients showed increasing levels of IL‐4 and IL‐10 with a combined vigorous‐aerobic and strength exercise regimen compared to sedentary controls 40. Similarly, a 6‐month RCT in diabetic patients 48 and an 8‐week RCT in postmyocardial infarction patients 44 showed moderate‐aerobic exercise increased IL‐10 levels compared to control groups. One study among healthy individuals, a small (*n* = 17) 8‐week low‐intensity aerobic exercise intervention, showed increased levels of circulating IL‐10 41. Regular vigorous intensity, interval training increased IL‐10 levels in a 6‐month RCT among angina patients 43. The studies reporting no effects on circulating anti‐inflammatory cytokines, include two RCTs of resistance training (12‐weeks [59] and 24‐weeks 61), an 8‐week intervention trial of combined strength and aerobic exercise regimen 62, and an 8‐month RCT of moderate‐aerobic activity 60. Among breast cancer survivors, RCTs of combined moderate‐aerobic and resistance training (8‐weeks 63 and 3‐months 64) showed no significant effects on IL‐10 63, 64, IL‐4 63, or other anti‐inflammatory cytokines (i.e., IL‐1ra) 63.

Our analyses of baseline characteristics as potential moderators were inconclusive with inconsistent results between the two trials. In BETA, significantly, higher postintervention levels of circulating IL‐10 in the high‐volume compared to moderate‐volume exercise groups were observed among less fit (VO_2max_ < 27.2 mL/kg/min) and older (aged >60 years) women, whereas significantly, lower postintervention levels were observed among more fit (VO_2max_ ≥27.2 mL/kg/min) and younger (aged ≤60 years) women at baseline. These mixed results may be due to chance, especially given the multiple testing and smaller numbers in the subgroup analyses.

ALPHA and BETA represent the first RCTs in healthy, postmenopausal women to examine the impact of a year‐long aerobic exercise, as well as the effect of different aerobic volumes at the same moderate intensity (150, 225, and 300 min per week at 70–80% of maximum effort) on anti‐inflammatory cytokines. Both ALPHA and BETA had excellent retention and adherence rates and both were well powered to detect differences between intervention groups [50, 51]. Fasting blood was collected after a 24‐h period of no exercise; thus, we were able to examine chronic effects rather than transient effects of exercise on circulating IL‐4 and IL‐10. The random allocation produced balanced groups with respect to potential confounders (dietary intake, cholesterol levels, arthritis, and use of nonsteroidal anti‐inflammatory) in both trials. An additional strength is our comprehensive approach with similar finding based on ITT, per‐protocol, and adherence analyses.

The limitations of our study are important to consider. Our levels of IL‐4 and IL‐10 were lower in BETA than ALPHA, which may reflect the assays being performed separately for ALPHA and BETA. We decided a priori to examine the exercise‐induced effect on IL‐1ra in addition to IL‐10 and IL‐4 based on the established elevated levels of these cytokines with acute exercise 21, 22, 23, 24. However, we were unable to include IL‐1ra in our analyses because of a low signal from our samples. Lastly, we did not adjust for multiple testing and recognize that some of our findings may be due to chance.

An inactive lifestyle leads to the accumulation of visceral fat and a proinflammatory phenotype of adipose tissue leading to a state of persistent low‐grade systemic inflammation 23. Given the substantial amount of evidence from observational and RCTs demonstrating physical activity effected reductions in proinflammatory biomarkers associated with chronic inflammation 14, 15, 16, 17, 18, 19, 20, further mechanistic studies are needed to clarify the potential anti‐inflammatory effects of regular physical activity and may require a system‐based or multidimensional approach versus individual markers [65]. For instance, the IL‐10 to TNF‐*α* ratio has been recently postulated as a marker for the assessment of the degree of inflammation and may increase with regular physical activity [66]. Since the magnitude of the exercise‐induced IL‐6 response is dependent on intensity and duration of the exercise [27], physical activity regimens incorporating high‐intensity intervals compared to continuous moderate‐intensity exercises may have additional benefit in mediating exercise‐induced anti‐inflammatory effects. An improved understanding of the anti‐inflammatory effects of physical activity and the biologically relevant dose (intensity and volume) has important implications for contributing to clinically meaningful evidence‐based recommendations for the prevention of cancers and other chronic diseases and indeed, there has been growing interest in examining the anti‐inflammatory effects of exercise on cardiovascular disease [67] and type 2 diabetes [67].

Overall, the results from ALPHA and BETA suggest that year‐long, moderate‐intensity physical activity regimens of various volumes do not have a significant impact on levels of the anti‐inflammatory markers, IL‐4 and IL‐10, among healthy, postmenopausal women.

**Clinicaltrials.gov identifier:** NCT00522262 (ALPHA) and NCT1435005 (BETA).

## Ethics Approval

Ethics approval for ALPHA was obtained from the Alberta Cancer Research Ethics Committee, the Health Research Ethics Board of the University of Alberta, and the Conjoint Health Research Ethics Board (CHREB) of the University of Calgary. Ethics approval for BETA was obtained from the Health Research Ethics Board of Alberta and CHREB.

## Availability of Data and Materials

The datasets supporting the conclusions of this article will not be shared due to ongoing analyses from ALPHA and BETA.

## Conflict of Interest

The authors do no declare any competing interests.

## Supporting information


**Table S1.** Per‐protocol analysis of anti‐inflammatory cytokine levels for exercisers and controls in the Alberta Physical Activity and Breast Cancer Prevention Trial (ALPHA) and high‐volume and moderate‐volume exercisers in the Breast Cancer and Exercise Trial in Alberta (BETA) at 6 and 12 months from baseline.

**Table S2.** Adherence level analyses of anti‐inflammatory cytokine levels at baseline and 12 months in exercisers and controls in the Alberta Physical Activity and Breast Cancer Prevention Trial (ALPHA) and high‐volume and moderate‐volume exercisers in the Breast Cancer and Exercise Trial in Alberta (BETA).

**Figure S1.** Levels of (A) IL‐4 and (B) IL‐10 at baseline, 6, and 12 months for exercisers and controls in the Alberta Physical Activity and Breast Cancer Prevention Trial (ALPHA) and high‐volume and moderate‐volume exercisers in the Breast Cancer and Exercise Trial in Alberta (BETA).
