# Supplementary material for: Impact of aerobic exercise on levels of IL‐4 and IL‐10: results from two randomized intervention trials
Source: Cancer Med. 2016 Aug 3;5(9):2385–97. doi: 10.1002/cam4.836 (PMC5055172; doi:10.1002/cam4.836)
Supplement: Supplementary file 1 — Table S1. Per‐protocol analysis of anti‐inflammatory cytokine levels for exercisers and controls in the Alberta Physical Activity and Breast Cancer Prevention Trial (ALPHA) and high‐volume and moderate‐volume exercisers in the Breast Cancer and Exercise Trial in Alberta (BETA) at 6 and 12 months from baseline. [file CAM4-5-2385-s001.docx]

**Supplementary Table S1.** Per-protocol analysis of anti-inflammatory cytokine levels for exercisers and controls in the ALPHA Trial and HIGH volume and MODERATE volume exercisers in BETA at 6 and 12 months from baseline

|  | **Baseline** |  | **6 Months** |  | **12 Months** | ***n*** | **Percent change from baseline to 12 months** | **TER of Exercise/Control or High/Moderate (95% CI)^b^** | **Between-Group P** |
| --- | --- | --- | --- | --- | --- | --- | --- | --- | --- |
|  | **Geometric mean (95% CI)^a^** |  | **Geometric mean (95% CI)^a^** |  | **Geometric mean (95% CI)^a^** |  |  |  |  |
| **ALPHA^c^** |  |  |  |  |  |  |  |  |  |
| IL-4 (pg/mL) |  |  |  |  |  |  |  |  |  |
| Exercisers | 1.35 (1.18, 1.56) |  | 1.33 (1.16, 1.52) |  | 1.33 (1.16, 1.53) | 97 | -1.84 | 1.00 (0.92, 1.07) | 0.92 |
| Control | 1.57 (1.40, 1.76) |  | 1.52 (1.35, 1.71) |  | 1.52 (1.35, 1.71) | 147 | -3.44 |  |  |
| IL-10 (pg/mL) |  |  |  |  |  |  |  |  |  |
| Exercisers | 1.30 (1.13, 1.48) |  | 1.26 (1.09, 1.44) |  | 1.21 (1.04, 1.39) | 97 | -7.11 | 0.96 (0.88, 1.04) | 0.28 |
| Control | 1.36 (1.22, 1.52) |  | 1.35 (1.19, 1.52) |  | 1.34 (1.19, 1.51) | 147 | -1.63 |  |  |
|  |  |  |  |  |  |  |  |  |  |
| **BETA^d^** |  |  |  |  |  |  |  |  |  |
| IL-4 (pg/mL) |  |  |  |  |  |  |  |  |  |
| High | 0.67 (0.52, 0.87) |  | 0.69 (0.54, 0.89) |  | 0.59 (0.45, 0.78) | 85 | -11.45 | 0.93 (0.78, 1.10) | 0.38 |
| Moderate | 0.88 (0.70, 1.10) |  | 0.91 (0.72, 1.15) |  | 0.83 (0.65, 1.05) | 116 | -5.54 |  |  |
| IL-10 (pg/mL) |  |  |  |  |  |  |  |  |  |
| High | 0.85 (0.72, 1.01) |  | 0.82 (0.69, 0.97) |  | 0.81 (0.68, 0.96) | 86 | -5.27 | 1.01 (0.92, 1.12) | 0.78 |
| Moderate | 0.90 (0.78, 1.04) |  | 0.85 (0.74, 0.98) |  | 0.83 (0.71, 0.97) | 116 | -7.87 |  |  |

^a^Of the 310 ALPHA and 386 BETA participants who provided blood samples at any time point, we excluded those with IL-4 or IL-10 levels above a threshold for extremely high levels, specifically: 10 pg/mL for both IL-4 (n=5 excluded) and IL-10 (n=5 excluded) in ALPHA; and 50 pg/mL for IL-4 (n=1 excluded) and 35 pg/mL for IL-10 (n=1 excluded) in BETA. Participants (n=8 for ALPHA and n=2 for BETA)missing a blood sample at any time point were also removed.

^b^The TER was calculated based on a linear mixed model for each cytokine, adjusted for time and baseline value. The TER represents the adjusted ratio of geometric means for the exercise group over the control group (ALPHA) or the high volume exercise group over the moderate volume exercise group (BETA). A TER of less than 1.0 indicates lower anti-inflammatory cytokine levels in the exercise group relative to the control group (ALPHA) or the high volume exercise group relative to the moderate volume exercise group (BETA) at 6 and 12 months; a TER greater than one indicates higher anti-inflammatory cytokines in the exercise group (ALPHA) or the high volume exercise group (BETA); and a TER of 1.0 indicates no differences between exercise groups compared.

^c^Per-protocol analysis of ALPHA includes those that adhered to at least 80% of the prescribed exercise (≥180min) over weeks 1-52 of the intervention.

^d^Per-protocol analysis of BETA includes those that adhered to 80-100% of the moderate volume exercise prescription (120-150min) and at least 80% (≥240min) of the high volume exercise prescription over weeks 1-52 of the intervention.

Abbreviations: CI, confidence interval; IL-4, interleukin-4; IL-10, interleukin-10; TER, treatment effect ratio.
