# Supplementary material for: Impact of aerobic exercise on levels of IL‐4 and IL‐10: results from two randomized intervention trials
Source: Cancer Med. 2016 Aug 3;5(9):2385–97. doi: 10.1002/cam4.836 (PMC5055172; doi:10.1002/cam4.836)
Supplement: Supplementary file 2 — Table S2. Adherence level analyses of anti‐inflammatory cytokine levels at baseline and 12 months in exercisers and controls in the Alberta Physical Activity and Breast Cancer Prevention Trial (ALPHA) and high‐volume and moderate‐volume exercisers in the Breast Cancer and Exercise Trial in Alberta (BETA). [file CAM4-5-2385-s002.docx]

**Supplementary Table S2.** Adherence level^a^ analyses of anti-inflammatory cytokine levels at baseline and 12 months in exercisers and controls in the ALPHA and high volume and moderate volume exercisers in the BETA.

|  | **Baseline** |  | **12 Months** |  | **Ratio 12 Months / Baseline (95% CI)^c^** | **Percent Change^d^** | ***P*^e^** | ***P*_trend_^f^** |
| --- | --- | --- | --- | --- | --- | --- | --- | --- |
|  | **Geometric Mean (95% CI)^b^** |  | **Geometric Mean (95% CI)^b^** | ***n*** |  |  |  |  |
| **ALPHA** |  |  |  |  |  |  |  |  |
| IL-4 (pg/mL) |  |  |  |  |  |  |  |  |
| Controls | 1.57 (1.40, 1.76) |  | 1.52 (1.35, 1.71) | 147 | 0.97 (0.91, 1.03) | -3.44 | Ref. | 0.62 |
| <150 min/wk | 1.46 (1.14, 1.87) |  | 1.37 (1.08, 1.72) | 38 | 0.94 (0.82, 1.07) | -6.39 | 0.53 |  |
| 150-225 min/wk | 1.41 (1.22, 1.63) |  | 1.35 (1.17, 1.56) | 92 | 0.96 (0.89, 1.04) | -4.22 | 0.62 |  |
| >225 min/wk | 1.09 (0.80, 1.49) |  | 1.09 (0.81, 1.45) | 20 | 1.00 (0.84, 1.18) | -0.40 | 0.80 |  |
| IL-10 (pg/mL) |  |  |  |  |  |  |  |  |
| Controls | 1.36 (1.22, 1.52) |  | 1.34 (1.19, 1.51) | 147 | 0.98 (0.93, 1.04) | -1.63 | Ref. | 0.68 |
| <150 min/wk | 1.38 (1.08, 1.77) |  | 1.40 (1.10, 1.77) | 38 | 1.01 (0.85, 1.21) | 1.33 | 0.68 |  |
| 150-225 min/wk | 1.29 (1.13, 1.47) |  | 1.25 (1.09, 1.44) | 91 | 0.97 (0.88, 1.07) | -3.00 | 0.70 |  |
| >225 min/wk | 1.01 (0.69, 1.48) |  | 1.01 (0.67, 1.52) | 21 | 1.00 (0.85, 1.17) | -0.05 | 0.80 |  |
|  |  |  |  |  |  |  |  |  |
| **BETA** |  |  |  |  |  |  |  |  |
| IL-4 (pg/mL) |  |  |  |  |  |  |  |  |
| <150 min/wk | 0.89 (0.76, 1.04) |  | 0.85 (0.72, 0.99) | 227 | 0.95 (0.88, 1.03) | -5.13 | Ref | 0.29 |
| 150-250 min/wk | 0.94 (0.75, 1.19) |  | 0.82 (0.65, 1.03) | 88 | 0.86 (0.74, 1.01) | -13.58 | 0.32 |  |
| >250 min/wk | 0.67 (0.51, 0.88) |  | 0.61 (0.45, 0.82) | 70 | 0.91 (0.74, 1.13) | -8.54 | 0.39 |  |
| IL-10 (pg/mL) |  |  |  |  |  |  |  |  |
| <150 min/wk | 0.84 (0.76, 0.94) |  | 0.79 (0.71, 0.88) | 227 | 0.94 (0.88, 0.99) | -6.44 | Ref | 0.88 |
| 150-250 min/wk | 0.89 (0.73, 1.07) |  | 0.81 (0.68, 0.96) | 88 | 0.91 (0.82, 1.02) | -8.63 | 0.77 |  |
| >250 min/wk | 0.82 (0.69, 0.97) |  | 0.77 (0.65, 0.91) | 70 | 0.94 (0.84, 1.04) | -6.50 | 0.94 |  |

^a^Adherence was calculated as the mean minutes of exercise per week over the 52 weeks of the study.

^b^ Of the 310 ALPHA and 386 BETA participants who provided a blood sample at any time point, we excluded those with IL-4 or IL-10 levels above a threshold for extremely high levels, specifically: 10 pg/mL for both IL-4 (n=5 excluded) and IL-10 (n=5 excluded) in ALPHA; and 50 pg/mL for IL-4 (n=1 excluded) and 35 pg/mL for IL-10 (n=1 excluded) in BETA. Participants (n=8 for ALPHA and n=2 for BETA) missing a blood sample at any time point were also removed.

^c^Ratio of geometric means at 12 months to geometric mean at baseline, adjusted for baseline values.

^d^Percentage change in adherence group mean of each inflammatory cytokine at 12 months from baseline, adjusted for baseline values.

^e^P-value tests difference in changes in inflammatory cytokine levels between the lowest adherence group and the specified adherence group, adjusted for the baseline value. A unified model, where the adherence group was treated as a categorical variable was used to calculate the p-values, which correspond to β-coefficients for the other groups, using the lowest group as the referent group.

^f^Trend across adherence groups was tested using a linear model predicting change from baseline to 12 months, adjusted for baseline biomarker values, where adherence category was treated as a continuous variable

Abbreviations: CI, confidence interval; IL-4, interleukin-4; IL-10, interleukin-10 ; TER, treatment effect ratio.
