# Supplementary material for: Impact of aerobic exercise on levels of IL‐4 and IL‐10: results from two randomized intervention trials
Source: Cancer Med. 2016 Aug 3;5(9):2385–97. doi: 10.1002/cam4.836 (PMC5055172; doi:10.1002/cam4.836)
Supplement: Supplementary file 3 — Figure S1. Levels of (A) IL‐4 and (B) IL‐10 at baseline, 6, and 12 months for exercisers and controls in the Alberta Physical Activity and Breast Cancer Prevention Trial (ALPHA) and high‐volume and moderate‐volume exercisers in the Breast Cancer and Exercise Trial in Alberta (BETA). [file CAM4-5-2385-s003.docx]

**Supplementary Figure S1.** Levels of (A) IL-4 and (B) IL-10 at baseline, 6 and 12 months for exercisers and controls in the ALPHA Trial and HIGH volume and MODERATE volume exercisers in the BETA
